# Impact of Preterm Birth on Neurodevelopmental Disorders in South Korea: A Nationwide Population-Based Study

**DOI:** 10.3390/jcm11092476

**Published:** 2022-04-28

**Authors:** Jong Ho Cha, Ja-Hye Ahn, Yun Jin Kim, Bong Gun Lee, Johanna Inhyang Kim, Hyun-Kyung Park, Bung-Nyun Kim, Hyun Ju Lee

**Affiliations:** 1 Department of Pediatrics, Hanyang University Hospital, Hanyang University College of Medicine, Seoul 04763, Korea; cjhchany@gmail.com (J.H.C.); mdscually@gmail.com (J.-H.A.); neopark@hanyang.ac.kr (H.-K.P.); 2 Biostatistical Consulting and Research Lab, Medical Research Collaborating Center, Hanyang University, Seoul 04763, Korea; yeun0148@hanyang.ac.kr; 3 Department of Orthopedic Surgery, Hanyang University Hospital, Hanyang University College of Medicine, Seoul 04763, Korea; orthdr@naver.com; 4 Clinical Research Institute of Developmental Medicine, Hanyang University Hospital, Seoul 04763, Korea; iambabyvox@hanmail.net; 5 Department of Psychiatry, Hanyang University Hospital, Hanyang University College of Medicine, Seoul 04763, Korea; 6 Division of Child and Adolescent Psychiatry, Department of Psychiatry, Seoul National University Hospital, Seoul 03080, Korea

**Keywords:** neurodevelopmental disorder, preterm birth, autism spectrum disorder, developmental delay, nationwide cohort

## Abstract

Neurodevelopmental disorder (NDD) in preterm infants has become of great interest. We aimed to investigate the impact of preterm birth on the proportion of NDD using nationwide data provided by the Korean National Health Insurance Service. We included 4894 extremely preterm or extremely low-birth-weight (EP/ELBW; <28 weeks of gestation or birth weight < 1000 g) infants, 70,583 other preterm or low-birth-weight (OP/LBW; 28–36 weeks of gestation or birth weight < 2500 g) infants, and 264,057 full-term infants born between 2008 and 2015. We observed their neurodevelopment until 6 years of age or until the year 2019, whichever occurred first. Diagnoses of NDDs were based on the World Health Organization’s International Classification of Diseases 10th revision. An association between preterm birth and NDD was assessed using a multivariable logistic regression model. There was a stepwise increase in the risk of overall NDD with increasing degree of prematurity, from OP/LBW (adjusted odds ratio 4.46; 95% confidence interval 4.34–4.58), to EP/ELBW (16.15; 15.21–17.15). The EP/ELBW group was strongly associated with developmental delay (21.47; 20.05–22.99), cerebral palsy (88.11; 79.89–97.19), and autism spectrum disorder (11.64; 10.37–13.06). Preterm birth considerably increased the risk of NDD by the degree of prematurity.

## 1. Introduction

Recent advances in neonatal care have led to a marked increase in the survival rate of preterm infants. According to the Korean Statistical Information Service in 2020, preterm birth (gestational age (GA) < 37 weeks) and low birth weight (LBW; birth weight < 2500 g) accounted for 8.6% and 6.8% of total births, respectively, both of which were markedly higher than the figures for a decade ago, 5.8% and 4.9% [1]. As the number of survivors of preterm birth increases, their long-term neurodevelopmental issues have become of interest. Unfortunately, despite advancements in perinatal care, the neurodevelopmental outcomes of preterm infants have not been markedly improved [2,3]. In Australia, the neurodevelopmental outcomes of extremely preterm (EP; <28 weeks of GA) survivors were evaluated at eight years of age and found to be stationary over the past decade [4]. A recent French nationwide cohort study showed that rates of moderate-to-severe and mild neurodevelopmental impairments were 28% and 38.5%, respectively, in the EP population at preschool ages [5].

Neurodevelopmental disorder (NDD) is a group of lifelong conditions characterized by impairments in cognitive, communication, behavior, and motor skills [6]. Growing evidence suggests preterm birth has a high risk of a wide spectrum of NDD, including autism spectrum disorder (ASD), attention-deficit hyperactivity disorder (ADHD), intellectual disability (ID), and language disorder (LD) [7,8,9,10,11]. Even preterm infants born without visible brain injuries may result in later neurodevelopmental impairment [12,13]. Thus, every preterm population is at risk of developing NDD, and this risk should be thoroughly investigated. However, there is a limited literature regarding the risk of a wide spectrum of NDD in the preterm populations using nationwide data.

As a definition of neurodevelopmental impairment varies across the nations or cohorts, definitions used in the study significantly influence the probability of NDD at diverse ages [14]. For instance, the incidence of neurodevelopmental impairment of EP survivors measured at 21 months of corrected age varied from 3.5% to 14.9% by the definition in a recent Canadian cohort study [15]. Moreover, the incidence of neurodevelopmental impairment is affected by the degree of prematurity. Prevalence of any neurodevelopmental impairment in EP survivors at two to five years of age is known to be substantial, as high as 11–37% in North America and 42% in the rest of the world [16]. For survivors with 28–31 weeks of GA, the incidence of neurodevelopmental disorder was 19% at five years of age [5]. In addition, the neurodevelopmental outcomes of the moderate-to-later preterm population are notable, reflecting the potential risk of the group [17]. Measured in two years of corrected age, the incidence of moderate-to-severe language delay was 13.7%, which was significantly higher than the term-born group in a previous Australian report [18]. Despite these methodological issues, the neurodevelopmental assessment of every preterm survivor is needed considering the high incidence of neurodevelopmental impairment.

Herein, we investigated the overall prevalence of NDD in preschool-age children who were born prematurely, stratified by demographic and socioeconomic characteristics, using the nationwide database provided by Health Insurance Review and Assessment Service (HIRA) in South Korea.

## 2. Materials and Methods

### 2.1. Data Source and Case Definition

We used the database obtained from the HIRA. The HIRA stores and reviews data on medical claims for the South Korean population, including diagnostic codes, medical visits, prescription records, and demographic information. In South Korea, each individual is registered with his/her health insurance identification number. More than 97% of the population is covered by national health insurance, and the remaining population is covered by the medical-aid program that provides healthcare services to low-income households. Thus, medical-aid beneficiaries can be defined as having the lowest socioeconomic status. Records with diagnostic codes were based on the World Health Organization’s International Classification of Diseases 10th revision (ICD-10). The study was approved by the Institutional Review Board of Hanyang University (IRB No. 2020-08-031). Informed consent was not required since public data from the HIRA were used.

We analyzed health claims data of the pediatric population with ICD-10 birth codes recorded between 2008 and 2019 in the HIRA database. Our study population included newborns from 2008 to 2015 and observed their NDD until six years of age or until the year 2019, whichever occurred first. Since the database contain missing data in the ICD-10 birth codes for GA or birth weight, the study population was subdivided into three subgroups combining these measures; (1) EP or extremely low-birth-weight (ELBW) infants, with GA < 28 weeks or birth weight < 1000 g; (2) other preterm (OP) or LBW infants, with GA < 37 weeks or birth weight < 2500 g; and (3) full-term infants (FT), infants with GA ≥ 37 weeks. Exclusion criteria were as follows: (1) children diagnosed with congenital malformations of the nervous system (Q00, anencephaly and similar malformations; Q01, encephalocele; Q02, microcephaly; Q03, congenital hydrocephalus; Q04, other congenital malformation of the brain), (2) children diagnosed with chromosomal anomaly (Q09); and (3) children with missing information for GA or birth weight.

The NDDs of interest included developmental delay (DD), cerebral palsy (CP), ASD, ADHD, LD, ID, and tic disorder (TD). Detailed information about ICD-10 codes for the definition of the study group and each sub-condition is summarized in Table 1. DD was diagnosed with children with a significantly delayed attainment of the expected physiological developmental stage. The diagnosis of NDD was made when children visited the outpatient clinic at least twice or those with more than one admission with a primary diagnosis. Each diagnosis was confirmed by clinical experts in pediatric rehabilitation, pediatric neurology, and pediatric psychiatry.

### 2.2. The Proportion of NDD

The annual cumulative incidence of each sub-condition was calculated starting on the 1 January of each year from 2012 to 2017. To avoid bias in investigating temporal trends of NDDs, we tried to contain populations with more than three different birth years in each year of diagnosis. The proportion of diagnosed cases based on the population subgroup in each sub-condition was calculated. The age of diagnosis was computed in years, since our data uses the age of diagnosis only in years, not in months.

### 2.3. Statistical Analysis

The prevalence of NDD was calculated by dividing the number of children diagnosed with NDD by the number of children who participated in the study. In addition, total cases were stratified by the degree of prematurity (categorized by individuals born EP/ELBW, OP/LBW, and FT), sex, and socioeconomic status (categorized by medium-to-highest and the lowest), and differences between each selected characteristic were evaluated. The age of diagnosis was found to be skewed, as assessed by the Anderson–Darling test, and was presented with interquartile ranges. A logistic regression model was used to estimate the adjusted odds ratio (aOR) and 95% confidence interval (C.I) to investigate significant factors for cases of NDD. The selected variables were the degree of prematurity, sex, and socioeconomic status. A univariable logistic regression analysis was performed to identify the association between the proportion of NDDs and preterm birth. A multivariable logistic regression analysis was performed after adjusting for sex and socioeconomic status. The probability of NDD based on age at the time of diagnosis were presented with Kaplan–Meier survival curves and compared among population subgroups using the log-rank test. Statistical analysis was performed using SAS version 9.4 (SAS Institute Inc., Cary, NC, USA).

## 3. Results

### 3.1. Demographics and the Proportion of NDD

**Figure 1:**
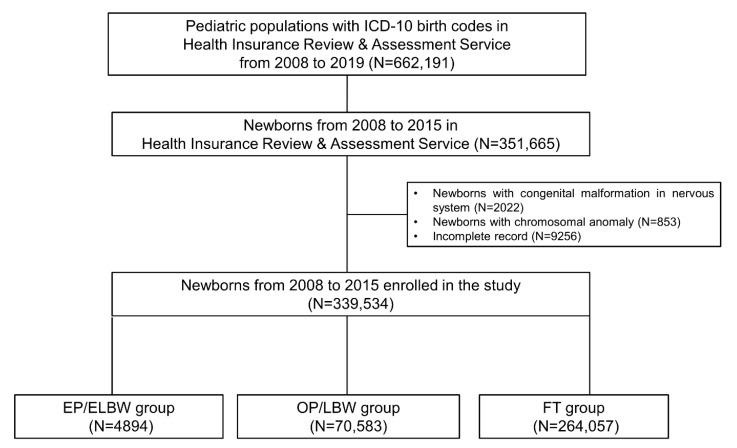
Flow chart of the study population. Abbreviations: ICD-10, the World Health Organization’s International Classification of Diseases 10th revision; EP, extremely preterm; ELBW, extremely low birth weight; OP, other preterm; LBW, low birth weight; FT, full-term.

Figure 1 summarizes the flow of the study population selection process. First, 662,191 pediatric populations with ICD-10 birth codes in the HIRA database were enrolled. Then, we selected 351,665 newborns who were born between 2008 and 2015. After exclusion criteria, 339,534 newborns were included in the study group; 4894 infants born EP/ELBW, 70,583 infants born OP, and 264,057 infants born FT.

The baseline demographic characteristics of children with NDD are summarized in Table 2. The prevalence of NDD in South Korea ranged from 0.6% to 4.1%, with that of DD and LD ranked 1 and 2, respectively, among the total population. In the prevalence of any NDD, males were more likely to be diagnosed than females. In socioeconomic status, except for TD, the lowest socioeconomic status group had more prevalent NDD cases than the medium-to-highest socioeconomic status group. Among sub-conditions, DD had the highest proportion of diagnosed cases, excluding CP, in both preterm populations, followed by ASD. CP, the most widely known NDD for preterm population, had the highest proportion of preterm infants than any other NDDs. Meanwhile, the median age of diagnosis differed among NDDs; indeed, DD, CP, and ASD were more prevalent before three years of age, while ADHD, LD, ID, and TD were more prevalent after three years of age. The annual cumulative incidence of NDD is summarized in Supplementary Table S1. Trends in the cumulative incidence also differed among the sub-conditions. In total, NDDs with decreasing trend over time was DD (0.76–0.58%), and CP (0.34–0.13%). On the other hand, the incidence of ADHD (0.03–0.32%), LD (0.28–0.47%), ID (0.06–0.14%), and TD (0.03–0.17%) increased and that of ASD (0.19–0.20%) was plateaued.

**Figure 2:**
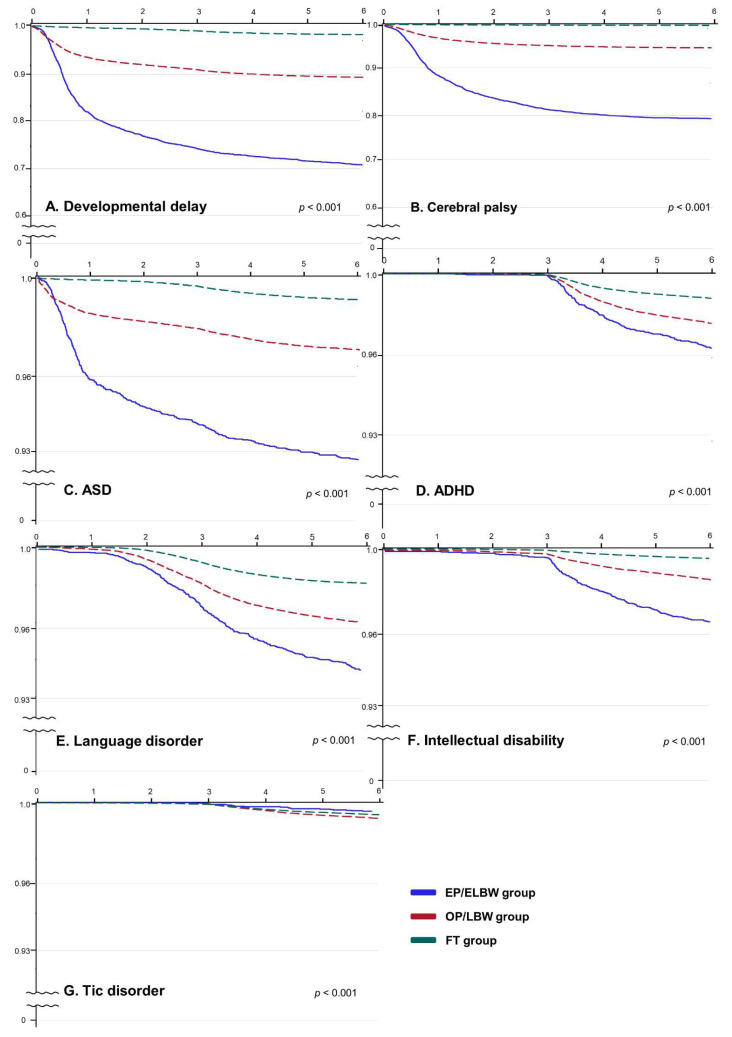
Comparisons of the probability of neurodevelopmental disorders in a pediatric population stratified by the degree of prematurity. All subjects are plotted against follow-up years until six years of age. The solid blue line represents the EP/ELBW group, the red dotted line represents the OP/LBW group, and the green dotted line represents the FT group. The x-axis represents follow-up time (year) and the y-axis represents the NDD-free probability. The log-rank test was applied, and *p* < 0.001 was considered statistically significant. Abbreviations: NDD, neurodevelopmental disorder; EP, extremely preterm; ELBW, extremely low birth weight; OP, other preterm; LBW, low birth weight; FT, full-term; ASD, autism spectrum disorder; ADHD, attention-deficit hyperactivity disorder.

The Kaplan–Meier survival curves for subgroups based on the age of diagnosis are presented in Figure 2. The NDD-free probability of each sub-condition was highest in the EP/ELBW group, followed by the OP/LBW and the FT group (*p* < 0.001). The NDD-free probability of DD and CP in both preterm groups was higher than that of the FT group at the beginning of the observation period; however, during the latter phase, the NDD-free probability of ADHD, LD, and ID in both preterm groups increased rapidly and was ultimately higher than that of the FT group.

### 3.2. Associations between Preterm Birth and NDD

Table 3 summarizes the result of the logistic regression model. The aOR of overall NDD was 4.46 (95% C.I 4.34–4.58) in the OP/LBW group and 16.15 (95% C.I 15.21–17.15) in the EP/ELBW group, respectively. The aOR of the EP/ELBW group was highest in CP (88.11; 79.89–97.19), followed by DD (21.47; 20.05–22.99) and ASD (11.64; 10.37–13.06). The rank of aOR in the OP/LBW group was as same as that of EP/ELBW group; however, the aOR of the OP/LBW group was smaller. The aORs of CP, DD, and ASD were 18.09 (95% C.I 16.74–19.54), 6.19 (95% C.I 5.97–6.43), and 4.11(95% C.I 3.86–4.38), respectively.

## 4. Discussion

This nationwide population-based study investigated the impact of preterm birth on NDD in South Korea. Our study revealed that preterm birth is an evident risk factor for NDD; the odds of an overall diagnosis were 16.15 times higher in the EP/ELBW group and 4.46 times higher in the OP/LBW group. In addition, the preterm population accounted for a considerable proportion of diagnosed cases. Lastly, preterm birth had the most significant impact on ASD and DD, following CP.

To the best of our knowledge, this is the first study in Asian countries to address the association between preterm birth and a wide spectrum of NDD using nationwide birth cohort data. Although there is likely an inflated preterm population who were monitored more closely with earlier diagnosis of NDD than children born with FT, our findings provide a valuable understanding of the national epidemiology of preterm infants with NDD in South Korea. Nevertheless, our study has several limitations which are associated with the characteristics of the raw data. First, as we selected study populations who assigned the birth codes of ICD-10, selection bias can occur. It is not obligatory for clinicians to assign birth codes regarding both GA and birth weight. To obtain as many individuals as possible, we entered subjects who assigned either one of the information. Second, we limited the age of interest to 0–6 years. Newly diagnosed cases or individuals diagnosed with one or more morbidities later than six years of age could not be calculated. Additionally, the observed time period among individuals varied from three to six years. Therefore, a gap between our findings and actual incidences might exist. Third, we could not consider clinical (i.e., mechanical ventilation, surfactant use) and environmental information (i.e., maternal medication, familial structure) which are not registered in the form of ICD-10 codes. Additionally, our study design may have overlooked co-morbidity cases and sub-clinical neurodevelopmental problems. Lastly, we could not consider individuals with undernutrition, and growth restrictions.

Risk factors associated with NDD are not yet completely understood and are expected to be multifactorial [19]. Regarding socioeconomic status, nationwide studies conducted in Taiwan [20] and in the USA [21] found that a lower socioeconomic status decreased the probability of NDD, which was contrary to our result. These contradictory findings may be implicated in the limited access to adequate medical services and the caregiver’s inability to recognize early signs of NDD. In South Korea, the government has implemented a ‘National Health Screening Program for infants and children’ since 2007, which consists of seven annual health check-ups until preschool age [22]. We suspect that this policy enabled early detection of NDD in the high-risk group, even in the population group with low socioeconomic status. However, given that a ‘medical blind spot’ still exists for health insurance services, the risk of NDD in the lowest socioeconomic status group could be underestimated.

Previous studies have shown that preterm birth is a significant risk factor for NDD. Recent meta-analysis studies have shown that the preterm population has as much as a three-fold increase in the aOR of NDD compared to the control group [7,17,23]. Our study is an up-to-date nationwide study with the current status and trend of NDD. Previous nationwide studies measured neurodevelopmental outcomes with the diagnosis of CP for motor impairment, the result of the Bayley Scales of Infant and Toddler test for cognitive and language impairment in toddler age [14]. At preschool age or older, intelligent quotient, academic achievement, and the questionnaires by parents were widely used [4,21]. Compared to those studies, our findings would be more conservative. Since we only confirmed diagnoses made by clinicians, subclinical neurodevelopmental difficulties (i.e., academic performance, peer relationship) can be overlooked. A few nationwide studies have been conducted using diagnostic codes as an evaluation method [8,10,24,25,26]. Compared to those studies, our study covers an up-to-date preterm population reflecting timely detection of long-term issues following recent better survival rates and the increasing number of preterm.

CP, which marked the highest aOR among NDDs, is a widely known sequela of preterm birth. Based on a nationwide study in South Korea [27], DD, ASD, and LD were the sub-conditions that most contributed to the recently increasing trend of NDD. We assume that preterm birth has considerable implications. In South Korea, DD (R62.0) is a diagnostic code for referral to tertiary hospitals when a delay in acquiring developmental skills is highly suspected for children < 3 years of age. It includes children in states who have not been diagnosed with the specific NDD and children with caregivers who avoid a certain diagnosis for reasons such as social myths and insurance issues. Thus, the decreasing probability of DD in the preterm population should not be interpreted as an improvement in the developmental outcome; rather, it suggests an emerging trend of early intervention with a precise evaluation of preterm infants with suspected DD.

In addition, considering that DD has high comorbidity to ASD [28], it should be noted that a high risk of DD can be attributable to the risk of ASD. In 2011, the ASD diagnosis in the Diagnostic and Statistical Manual of Mental Disorders Fifth Edition was revised due to its ambiguity. After revision, the symptom definition of ASD narrowed and its incidence is expected to be lower in the toddler group [29,30]. In particular, the probability of ASD in preterm infants was higher compared to a study performed in previous decades [31]. Differences may have originated from methodological differences since we selected cases of NDD in which the diagnostic code concerning birth (preterm or FT) was used at baseline. We excluded NDD cases without information regarding GA or birth weight to quantify the prevalence of NDD based on the degree of prematurity. Moreover, there is a tendency that the diagnostic code for the FT group (Z38) is not entered when perinatal issues occur (e.g., asphyxia, transient tachypnea of newborn, jaundice). Therefore, our study design could overlook those affected populations and have a relatively large proportion of preterm infants. Although the current analysis showed a decreasing prevalence of ASD in both preterm groups, caution must be exercised when interpreting the results. However, the results show that the probability of ASD has increased over time and recently plateaued. For ASD and LD, the median age at the time of diagnosis was higher than that of DD, suggesting that early identification of these disorders is still challenging. Our group has recently reported, with the use of neuroimaging, that preterm infants have alterations in fronto-limbic circuitry maturation, especially the cingulum, which is related to the core symptoms of ASD [32,33].

In addition, the preterm population showed an increasing trend and significant impact on both ADHD and LD. This was in line with a previous Swedish nationwide study, which showed that the risk of ADHD was inversely related to GA [8]. As shown in the median age of diagnosis, both ADHD and LD are diagnosed when higher cortical functions develop to a specific level, which implies that such disorders would become more prominent as children reach school age. Our findings suggest that increasing awareness of ADHD and LD is needed, since surviving preterm infants are more likely to present more issues at school age [34]. Unlike other NDDs we studied, the pattern of TD was different; highest in OP/LBW infants, followed by FT and EP/ELBW infants. The mean onset of TD is between six and seven years and the annual average prevalence is known to be 0.2–0.3% [35,36]. Therefore, it is hard to say that the OP/LBW or FT infants have a significant risk of TD compared to EP infants, based on our data. Further study with sufficient follow-up duration is needed.

## 5. Conclusions

Our study examined the prevalence and trend of NDD and the impact of preterm birth using nationwide data. The risk of NDD was considerably increased by the degree of prematurity, and this tendency was more prominent in ASD and DD. We emphasize the importance of NDD as preterm birth-related morbidity, and further intervention and management programs should be implemented.

## Figures and Tables

**Table 1:** ICD-10 diagnostic codes included to the definition of study groups and neurodevelopmental disorders.

Definition	ICD-10 Codes
EP/ELBW group ^a^	P07.0 (P07.00, P07.01, P07.02) P07.2 (P07.20, P07.21, P07.22, P07.23, P07.24, P07.25)
OP/LBW group ^b^	P07.1 (P07.10, P07.11, P07.12, P07.13, P07.14) P07.3 (P07.30, P07.31, P07.32)
FT group	Z38 (Z38.0 Z38.3 Z38.6)
Developmental delay ^c^	R62.0
Cerebral palsy	G8 (G80, G81, G82, G83)
Autism spectrum disorder	F84 (F84.0, F84.1, F84.2, F84.3, F84.4, F84.5, F84.8, F84.9)
Attention-deficit hyperactivity disorder	F90 (F90.0, F90.1, F90.8, F90.9)
Language disorder	F80 (F80.0, F80.1, F80.2, F80.3, F80.8, F80.9)
Intellectual disability	F7 (F70, F71, F72, F73, F78, F79)
Tic disorder	F95 (F95.0, F95.1, F95.2, F95.8, F95.9)

^a^ Contains preterm infants with P07.0 (extremely low birth weight; birth weight of less than 1000 g) and P07.2 (extremely preterm; <28 weeks of gestation). ^b^ Contains preterm infants with P07.1 (low birth weight; birth weight of less than 2500 g) and P07.3 (very preterm and moderate-to-later preterm; 28–36 weeks of gestation). ^c^ Diagnosed in children with a significant delay in acquiring developmental skill areas including gross motor, fine motor, verbal speech, language, and self-help. Abbreviations: ICD-10, the World Health Organization’s International Classification of Diseases 10th revision; EP, extremely preterm; ELBW, extremely low birth weight; OP, other preterm; LBW, low birth weight; FT, full-term.

**Table 2:** Baseline demographic findings of children diagnosed with neurodevelopmental disorders.

	DD	CP	ASD	ADHD	LD	ID	TD
Population subgroup, N (%)							
Total, 339,534 (100)	13,872 (4.1)	5494 (1.6)	4310 (1.3)	4551 (1.3)	7189 (2.1)	2268 (0.7)	2184 (0.6)
EP/ELBW group, 4894 (1.4)	1405 (28.7)	1023 (20.9)	370 (7.6)	160 (3.3)	284 (5.8)	161 (3.3)	22 (0.5)
OP/LBW group, 70,583 (20.8)	7516 (10.7)	3676 (5.2)	2056 (2.9)	1538 (2.2)	2433 (3.5)	974 (1.4)	564 (0.8)
FT group, 264,057 (77.8)	4951 (1.9)	795 (0.3)	1884 (0.7)	2853 (1.1)	4472 (1.7)	1133 (0.4)	1598 (0.6)
Sex, N (%)							
Male, 175,328 (51.6)	8459 (4.8)	3149 (1.8)	2924 (1.7)	3640 (2.1)	5138 (2.9)	1583 (0.9)	1574 (0.9)
Female, 164,206 (48.4)	5413 (3.3)	2345 (1.4)	1386 (0.8)	911 (0.6)	2051 (1.3)	685 (0.4)	610 (0.4)
Socioeconomic status, N (%)							
Medium-to-highest, 336,364 (99.1)	13,691 (4.1)	5409 (1.6)	4232 (1.3)	4422 (1.3)	7020 (2.1)	2158 (0.6)	2167 (0.6)
Lowest, 3170 (0.9)	181 (5.7)	85 (2.7)	78 (2.5)	129 (4.1)	169 (5.3)	110 (3.5)	17 (0.5)
The proportion of the diagnosed cases, %							
EP/ELBW group	10.1%	18.6%	8.6%	3.5%	4.0%	7.1%	1.0%
OP/LBW group	54.1%	66.9%	47.7%	33.8%	33.8%	43.0%	25.8%
FT group	35.8%	14.5%	43.7%	62.7%	62.2%	49.9%	73.2%
Median age of diagnosis	1.11 (0.41–2.92)	0.84 (0.43–1.75)	2.59 (0.64–3.77)	3.83 (3.41–4.60)	3.14 (2.47–3.96)	3.87 (3.25–4.82)	3.91 (3.28–4.71)

Data are expressed as number (%) for categorical variables and median [Q1–Q3] for continuous variables. Abbreviations: DD, developmental delay; CP, cerebral palsy; ASD, autism spectrum disorder; ADHD, attention-deficit hyperactivity disorder; LD, language disorder; ID, intellectual disability; TD, tic disorder; EP, extremely preterm; ELBW, extremely low birth weight; OP, other preterm; LBW, low birth weight; FT, full-term.

**Table 3:** Multivariable logistic regression model presenting the association between the probability of neurodevelopmental disorders and preterm birth adjusted for sex and socioeconomic status.

	Univariable		Multivariable	
	Odds Ratio	95% C.I	Odds Ratio	95% C.I
**DD**				
EP/ELBW group	21.08 ^a^	19.69–22.56	21.47 ^a^	20.05–22.99
OP/LBW group	6.24 ^a^	6.01–6.47	6.19 ^a^	5.97–6.43
FT group	reference		reference	
**CP**				
EP/ELBW group	87.51 ^a^	79.35–96.52	88.11 ^a^	79.89–97.19
OP/LBW group	18.19 ^a^	16.84–19.65	18.09 ^a^	16.74–19.54
FT group	reference		reference	
**ASD**				
EP/ELBW group	11.39 ^a^	10.15–12.78	11.64 ^a^	10.37–13.06
OP/LBW group	4.18 ^a^	3.92–4.45	4.11 ^a^	3.86–4.38
FT group	reference		reference	
**ADHD**				
EP/ELBW group	3.09 ^a^	2.63–3.64	3.20 ^a^	2.72–3.77
OP/LBW group	2.04 ^b^	1.91–2.17	1.98 ^b^	1.86–2.11
FT group	reference		reference	
**LD**				
EP/ELBW group	3.58 ^a^	3.16–4.05	3.67 ^a^	3.24–4.15
OP/LBW group	2.07 ^b^	1.97–2.18	2.03 ^b^	1.93–2.13
FT group	reference		reference	
**ID**				
EP/ELBW group	7.90 ^a^	6.68–9.33	8.07 ^a^	6.81–9.54
OP/LBW group	3.25 ^b^	2.98–3.54	3.16 ^b^	2.90–3.45
FT group	reference		reference	
**TD**				
EP/ELBW group	0.74	0.48–1.13	0.76	0.42–1.15
OP/LBW group	1.32 ^a^	1.20–1.46	1.30 ^a^	1.18–1.43
FT group	reference		reference	
**Overall**				
EP/ELBW group	15.40 ^a^	14.51–16.33	16.15 ^a^	15.21–17.15
OP/LBW group	4.48 ^a^	4.36–4.60	4.46 ^a^	4.34–4.58
FT group	reference		reference	

Abbreviations: C.I, confidence interval; EP, extremely preterm; ELBW, extremely low birth weight; OP, other preterm; LBW, low birth weight; DD, developmental delay; CP, cerebral palsy; ASD, autism spectrum disorder; ADHD, attention-deficit hyperactivity disorder; LD, language disorder; ID, intellectual disability; TD, tic disorder. ^a^ *p* < 0.001, ^b^ 0.001 < *p* < 0.05.

## Data Availability

The data presented in this study are available on request from the corresponding author.

## Supplementary Materials

The following supporting information can be downloaded at: https://www.mdpi.com/article/10.3390/jcm11092476/s1, Supplementary Table S1: Trends in annual cumulative incidence of neurodevelopmental disorders in the pediatric population from 2012 to 2017 in South Korea.
