# Supplementary material for: Impact of Preterm Birth on Neurodevelopmental Disorders in South Korea: A Nationwide Population-Based Study
Source: J Clin Med. 2022 Apr 28;11(9):2476. doi: 10.3390/jcm11092476 (PMC9099635; doi:10.3390/jcm11092476)
Supplement: Supplementary file 1 [file jcm-11-02476-s001.zip › jcm-1617176-supplementary.pdf]

Supplementary Table S1. Trends in annual cumulative incidence of neurodevelopmental disorders in the pediatric population from 2012 to 2017 in South Korea

**EP/ELBW group [a]**								
**Year**	**Person year**	**DD**	**CP**	**ASD**	**ADHD**	**LD**	**ID**	**TD**
2012	2,738	5.70	4.09	1.57	0	0.84	0.22	0.04
2013	3,425	6.04	3.83	1.58	0.03	0.76	0.29	0.03
2014	3,653	5.20	3.61	1.73	0.27	1.01	0.36	0.03
2015	3,989	5.37	3.69	1.18	0.40	0.90	0.43	0.05
2016	3,538	4.55	3.19	0.82	0.42	1.02	0.62	0.06
2017	2,880	3.26	1.63	0.69	1.08	1.08	0.97	0.17
Overall	20,223	5.05	3.37	1.27	0.36	0.95	0.47	0.06
**OP/LBW group [b]**								
**Year**	**Person year**	**DD**	**CP**	**ASD**	**ADHD**	**LD**	**ID**	**TD**
2012	37,379	2.23	0.93	0.46	0.04	0.56	0.12	0.04
2013	47,778	2.01	0.82	0.45	0.07	0.57	0.12	0.05
2014	53,281	1.96	0.77	0.48	0.11	0.58	0.16	0.09
2015	58,552	1.97	0.70	0.29	0.20	0.43	0.17	0.08
2016	50,494	1.50	0.73	0.33	0.40	0.62	0.25	0.15
2017	42,819	1.11	0.46	0.31	0.56	0.74	0.31	0.21
Overall	290,303	1.80	0.73	0.38	0.23	0.58	0.19	0.10
**FT group [c]**								
**Year**	**Person year**	**DD**	**CP**	**ASD**	**ADHD**	**LD**	**ID**	**TD**
2012	116,076	0.17	0.06	0.07	0.03	0.18	0.03	0.03
2013	159,550	0.21	0.05	0.07	0.03	0.23	0.03	0.03
2014	189,330	0.29	0.06	0.08	0.04	0.28	0.04	0.06
2015	232,138	0.31	0.04	0.08	0.08	0.23	0.05	0.06
2016	211,448	0.36	0.06	0.13	0.14	0.30	0.08	0.11
2017	184,280	0.41	0.04	0.17	0.25	0.34	0.09	0.16
Overall	1,092,822	0.30	0.05	0.10	0.10	0.28	0.05	0.08
**Total**								
**Year**	**Person year**	**DD**	**CP**	**ASD**	**ADHD**	**LD**	**ID**	**TD**
2012	156,193	0.76	0.34	0.19	0.03	0.28	0.06	0.03
2013	210,753	0.72	0.29	0.18	0.04	0.31	0.05	0.03
2014	246,264	0.72	0.26	0.19	0.06	0.36	0.07	0.06
2015	294,679	0.70	0.22	0.13	0.11	0.28	0.08	0.06
2016	265,480	0.63	0.23	0.18	0.19	0.37	0.12	0.11
2017	229,979	0.58	0.13	0.20	0.32	0.47	0.14	0.17
Overall	1,403,348	0.68	0.24	0.18	0.13	0.35	0.09	0.08

Incidence was presented as %

[a] Defined as newborns with gestational age < 28 weeks or birth weight < 1,000 g.

[b] Defined as newborns with gestational age < 37 weeks or birth weight < 2,500 g.

[c] Defined as newborns with gestational age ≥ 37 weeks.

Abbreviations: EP, extremely preterm; ELBW, extremely low birth weight; OP, other preterm; LBW, low birth weight; DD, Developmental delay; CP, Cerebral palsy; ASD, Autism spectrum disorder; ADHD, Attention-deficit hyperactivity disorder; LD, Language disorder; ID, Intellectual disability; TD, Tic disorder.
